# Association between 5-HT1A receptor C-1019G, 5-HTTLPR polymorphisms and panic disorder: a meta-analysis

**DOI:** 10.18632/aging.206087

**Published:** 2024-08-28

**Authors:** Wenli Zhu, Yangying Bu, Lijuan Wu, Junwei Li, Chuanfu Song, Yihui Hao

**Affiliations:** 1 Department of Psychiatry, Fourth Hospital of Wuhu City, Wuhu, Anhui, China; 2 Department of Psychiatry, Shenyang Mental Health Center, Shenyang, Liaoning, China; 3 Department of Psychiatry, First Affiliated Hospital of Zhengzhou University, Zhengzhou, Henan, China

**Keywords:** serotonin, 5-HT1a receptor, serotonin plasma membrane transport proteins, genetic polymorphism, panic attack

## Abstract

HTR1A C-1019G polymorphism (rs6295) and serotonin transporter promoter polymorphism (5-HTTLPR) have been linked with panic disorder (PD) in different ethnic backgrounds. Both these polymorphisms are in the promoter regions. However, results are inconsistent and contrasting evidence makes reliable conclusions even more challenging. A meta-analysis was conducted to test whether C-1019G polymorphism and 5-HTTLPR were involved in the etiology of PD. Articles researching the link between C-1019G, 5-HTTLPR polymorphisms, and PD were retrieved by database searching and systematically selected on the basis of selected inclusion parameters. 21 studies were included that examined the relationship of rs6295,5-HTTLPR polymorphisms with PD risk susceptibility (rs62957 polymorphism – 7 articles, and 5-HTTLPR polymorphism - 14 articles). A significant association was seen between the rs6295 polymorphism and PD pathogenesis, especially in Caucasian PD patients. No significant genetic linkage was found between the 5-HTTLPR polymorphism and PD. C-1019G polymorphism was involved in the etiology of PD in Caucasian patients. The 5-HTTLPR polymorphism was not a susceptibility factor of PD.

## INTRODUCTION

Panic disorder (PD) is an anxiety disorder that is characterized by unpredicted and recurrent panic attacks [1]. PD affects up to 4% of the general populace [2]. Previous studies have shown that genetic factors have a critical function in the pathobiology of PD since genetic causes are responsible for 43% of the variations observed in PD [3].

The selective serotonin-reuptake inhibitors (SSRIs) are the primary antidepressants for the treatment of PD [4–6]. Their mechanism of action is to inhibit 5-HT uptakes in the presynaptic cleft, thereby raising the level of available 5-HT in the synaptic cleft. Thus, polymorphisms in the genes that modulate serotonin level and affect 5-HT neurotransmission signal transduction, such as serotonin transporter (5-HTT) and 5-HT1A receptor (5-HTR1A) may be involved in the etiopathogenesis of PD.

On comparing positron emission tomography (PET) results with control subjects, patients suffering from PD had significantly lower 5-HT1A radioligand binding in select areas of the brain [7]. Further, 5-HT1A receptor gene was also found to be implicated in PD [8, 9]. *In vivo* studies showed that mice with 5-HT1A receptor gene knockout displayed higher anxiety-like symptoms relative to wild-type mice [10]. Therefore, the 5-HT1A receptor gene can be potentially involved in PD [11]. C-1019G single nucleotide polymorphism (SNP rs6295) is situated in the HTR1A promoter, and has been linked with several psychiatric diseases and differences in treatment response to antidepressants [12–14].

The human serotoninergic transporter gene (5-HTT) maps on chromosome 17q11.1-q12 [15] and modulates serotonin reabsorption from the synaptic cleft, thereby terminating the serotonergic system. Among the different 5-HTT gene single nucleotide polymorphisms, there is a single nucleotide polymorphism (44 bp insertion/deletion) in the 5-HTT promoter region – that result in two alleles (L-long and S-short) – has been extensively studied. The transcriptional activity associated with the L allele is significantly more efficient compared with that of the S allele [15, 16]. Therefore, the L allele displays higher serotonin reuptake and lower level of serotonin in the synaptic cleft, which increases the risk of the development of psychiatric disorders such as depression, anorexia nervosa, suicide ideation, and PD [17–20].

Previous case-control studies have explored the genetic association of 5-HT1A receptor C-1019G, 5-HTTLPR, and PD, but the results were contradictory and inconclusive mainly due to different ethnic-dependent backgrounds, false-positive results, and insufficient sample sizes. To overcome the limitations of previous studies, a meta-analysis was performed to identify the HTR1A and 5-HTT genetic SNPs in PD. We combined the results of different studies, studies with small sample sizes, and/or conflicting results, thereby increasing their statistical power than that of the individual studies.

## RESULTS

There was a total of 530 articles about 5-HT1A with PD in the database. After screening, 7 articles, including 967 Panic Attack Cases Groups and 999 healthy control Groups [8, 9, 21–25] were included in the meta-analysis (Table 1 and Supplementary Table 1).

**Table 1:** Summary of studies examining the relationship between the 5-HT1A C-1019G polymorphism and PD.

**References**	**Race**	**Results**	**Genotype**						**Allele**			
			**Case (n)**			**Control (n)**			**Case (n)**		**Control (n)**	
			**GG**	**GC**	**CC**	**GG**	**GC**	**CC**	**G**	**C**	**G**	**C**
Huang et al. (2004)	Caucasian	No association	24	43	20	23	50	34	91	83	96	118
Rothe et al. (2004)	German	G is associated with PD	42	59	32	27	70	37	143	123	144	124
Carolina et al. (2010)	German	G is associated with PD	33	53	21	24	62	39	119	95	110	140
Choi et al. (2010)	Korean	No association	44	43	7	58	46	7	131	57	162	60
Choe et al. (2012)	Korean	No association	12	71	111	8	62	102	95	293	78	266
Takashi et al. (2017)	Japan	G is associated with PD	10	54	55	5	55	59	74	164	65	173
Zou et al. (2020)	Han	No association	138	83	12	138	82	11	359	107	358	104

**Figure 1:**
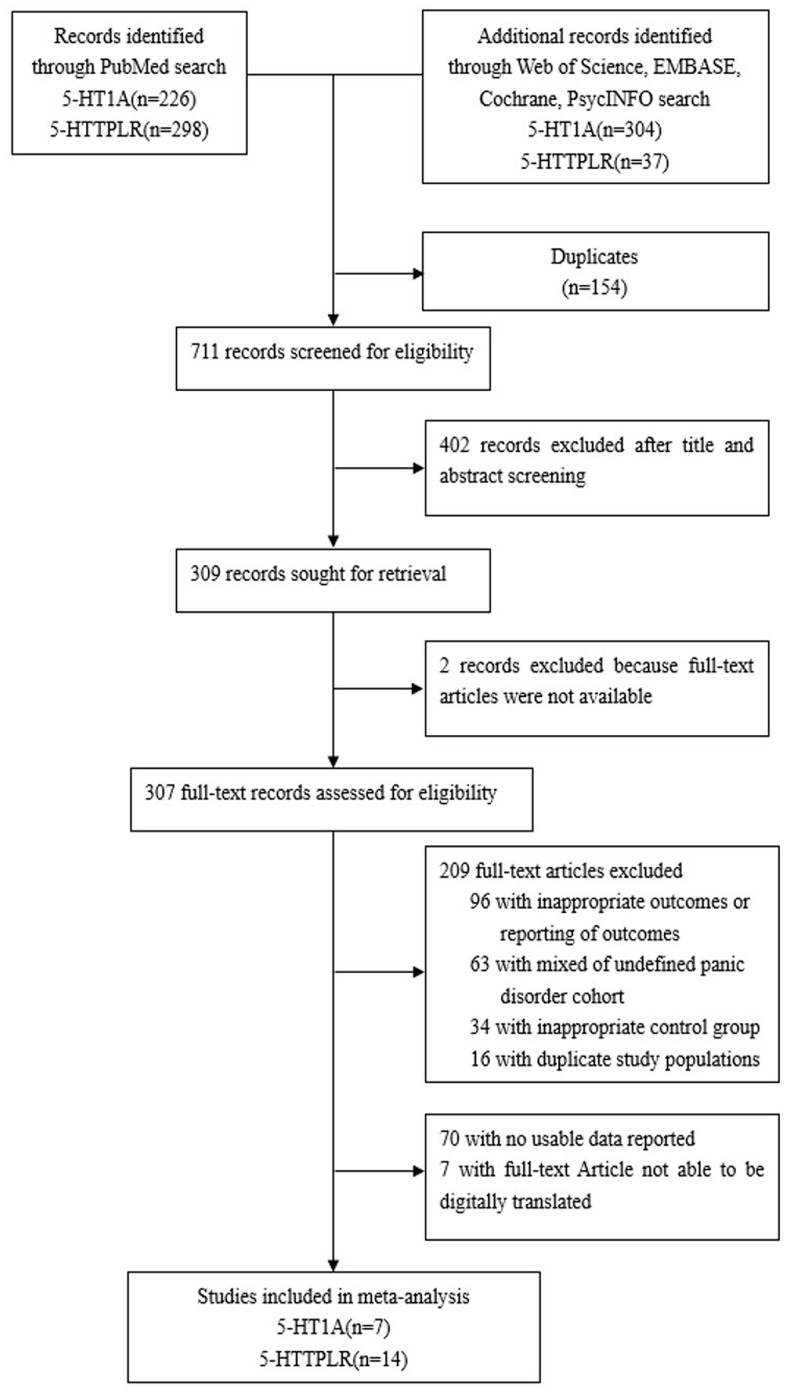
The detailed retrieval processes.

A total of 335 articles about 5-HTTPLR with PD were retrieved, excluding 107 duplicated articles, studies with missing data, animal studies, literature reviews, meeting abstracts, etc. After screening, 14 studies were selected (Table 2 and Supplementary Table 2). Among them, Deckert et al. [26] mentioned the case-control study data of the German population and the case-control study data of the Italian population. The data were included as two samples, and the total sample number was as follows: N = 15, [22, 24–36], see Figure 1 for the detailed retrieval process.

**Table 2:** The genotype frequency of 5-HTTPLR polymorphism and PD included studies.

**Author**	**Year**	**Race**	**Main results**	**Case**				**control**				**case**		**Control**	
				**Total**	**L/L**	**L/S**	**S/S**	**Total**	**L/L**	**L/S**	**S/S**	**L**	**S**	**L**	**S**
Deckert	1997	Germany	No association	85	29	44	12	90	32	42	16	102	68	106	74
Deckert	1997	Italian	No association	73	28	32	13	79	23	44	12	88	58	90	68
Ishiguro	1997	Japan	No association	66	2	13	51	150	4	32	114	17	113	40	260
Matsushita	1997	Japan	No association	86	7	35	44	213	10	78	125	49	123	98	328
Ohara	1999	Japan	No association	27	1	4	22	106	3	24	79	6	48	30	182
Samochowiec	2004	Poland	No association	95	45	40	10	202	85	97	20	130	60	264	140
Barrondo	2005	No mentioned	No association	92	32	42	18	174	64	74	36	87	97	202	146
Maron	2005	Estonia	No association	158	75	72	11	215	80	101	34	222	94	261	169
Olesen	2005	Denmark	No association	104	36	50	18	108	38	52	18	122	86	127	89
Kim	2006	Korea	No association	244	8	77	159	227	10	76	141	93	395	96	358
Choe	2013	Korea	No association	191	11	71	109	166	6	63	97	93	289	75	257
Watanabe	2017	Japan	No association	119	4	42	73	119	7	41	71	50	188	55	183
Schiele	2019	Caucasian	No association	109	36	59	14	536	207	263	66	131	87	692	395
Zou	2020	China	No association	233	22	63	148	231	20	90	121	107	359	130	332
Tanahashi	2021	Japan	No association	515	29	148	338	440	18	144	278	206	824	180	700

**Figure 2:**
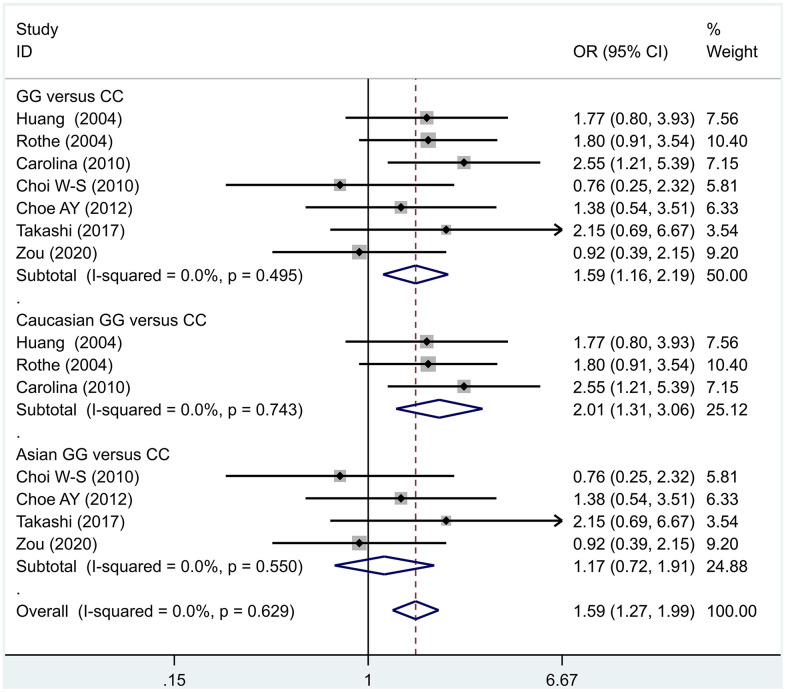
Results of the fixed-effects meta-analysis for the 5-HT1A genotype (GG versus CC) in the PD and control groups.

**Figure 3:**
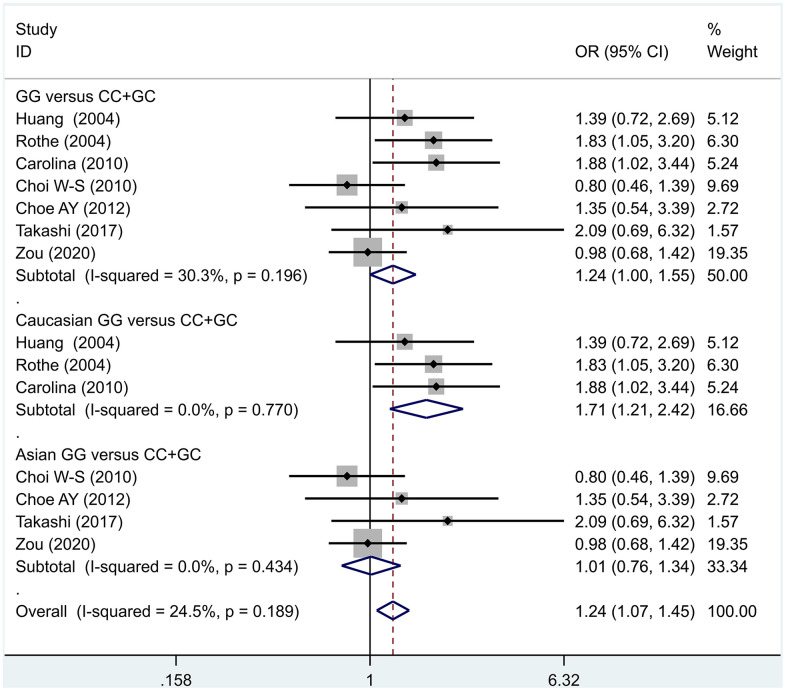
Results of the fixed-effects meta-analysis for the 5-HT1A genotype (GG versus CC +GC) in the PD and control groups.

**Figure 4:**
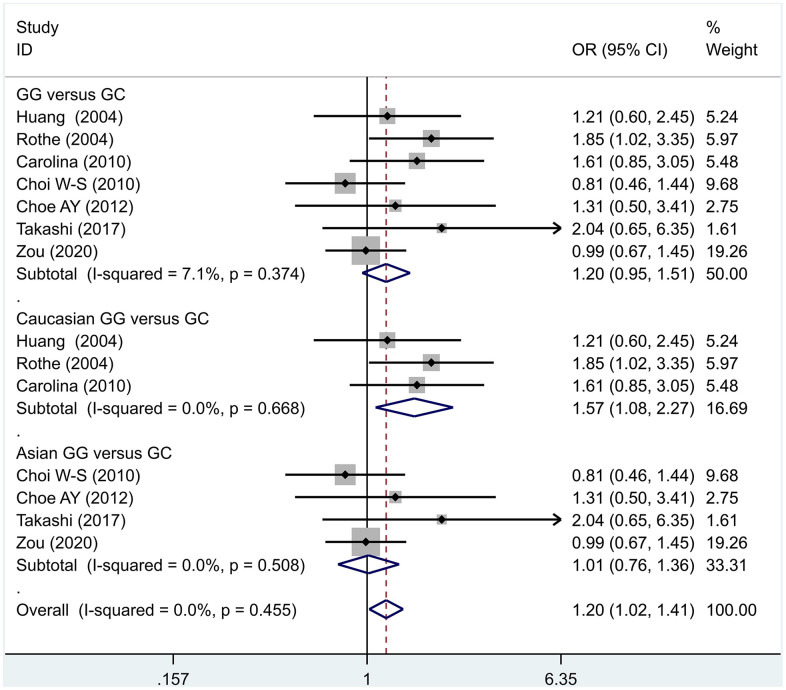
Results of the fixed-effects meta-analysis for the 5-HT1A genotype (GC versus CC) in the PD and control groups.

**Figure 5:**
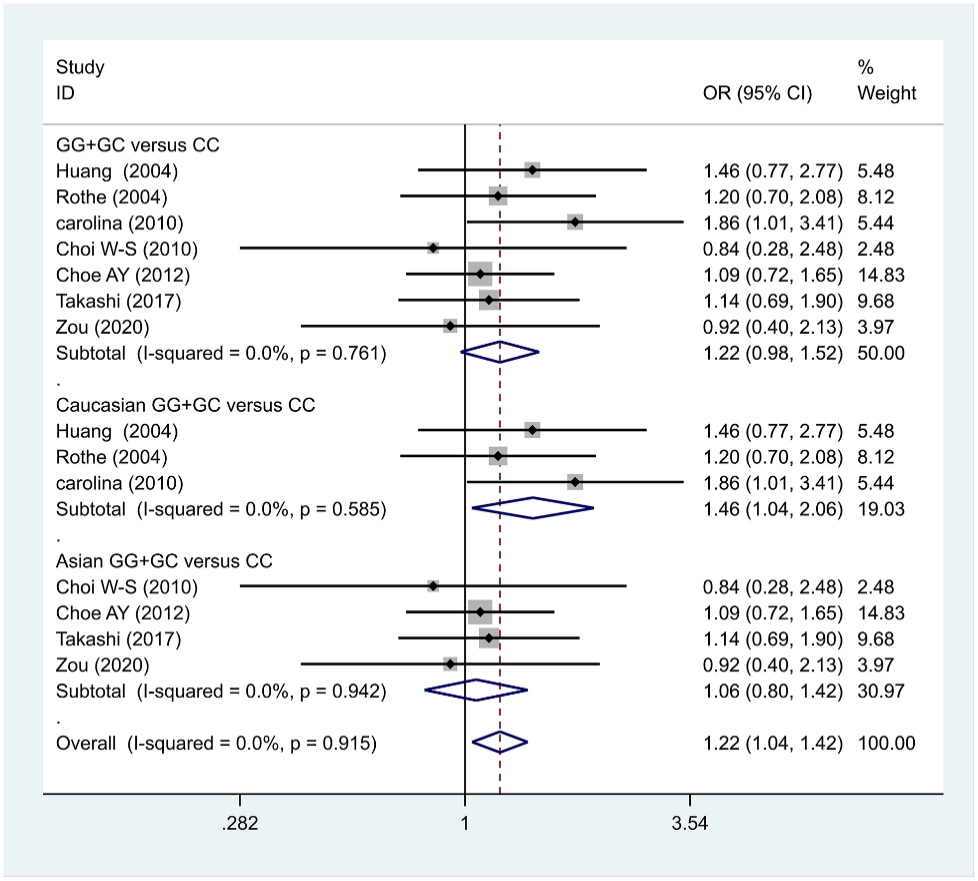
Results of the fixed-effects meta-analysis for the 5-HT1A genotype (GG+GC versus CC) in the PD and control groups.

**Figure 6:**
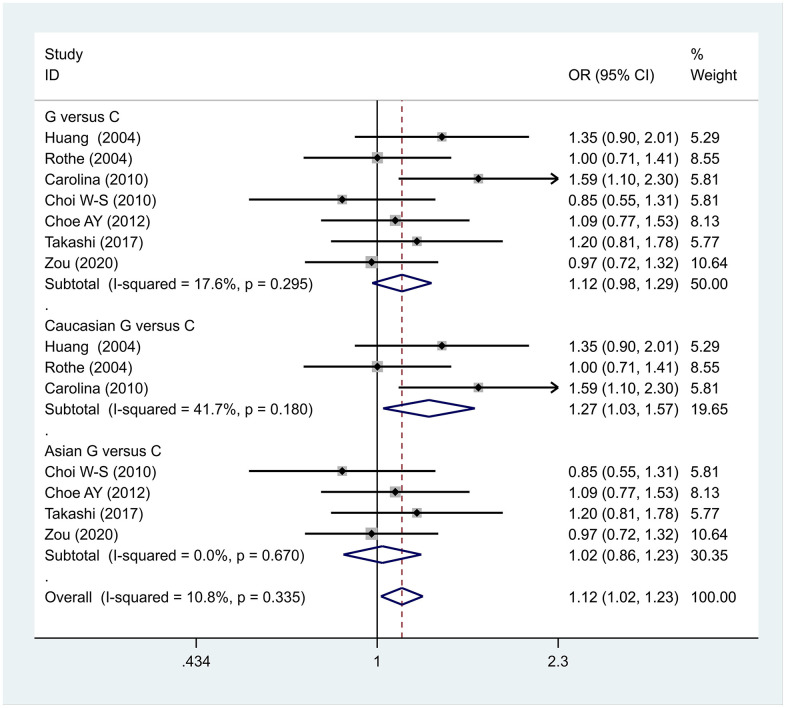
Results of the fixed-effects meta-analysis for the 5-HT1A allele (G vs. C) in the PD and control groups.

The data analysis results are shown in Figure 2. We analyzed the 7 articles about 5-HT1A with PD and constructed data models with G as the risk factor. A total of 5 data models were established (GG vs. CC, Figure 2, GG versus CC +GC, Figure 3, GC vs. CC, Figure 4, GG+GC vs. CC, Figure 5, G vs. C, Figure 6), no significant heterogeneity was found in all data models, and the fixed-effect model was employed for analyzing the results. The results of GG vs. CC analysis were OR = 1.59, 95%CI = (1.16-2.19), Z= 2.85, P = 0.004, Figure 2, GG versus CC+GC, OR = 1.24, 95%CI = (1.00-1.55) z = 1.97, P = 0.049, Figure 3. The results were statistically significant, and no positive results were found in other models. The results could be considered G allele as a risk factor for PD. Group analysis was conducted to address the differences between Asian and Caucasian races. The analysis results showed that OR = 2.00, 95%CI = (1.31-3.06), z = 3.22, P = 0.001, Figure 2. Caucasian GG versus CC+GC, OR = 1.71, 95%CI = (1.21-2.42) z = 3.02, p = 0.003, Figure 3. Caucasian G versus C, z= 2.21, P = 0.03, OR = 1.59, 95%CI = (1.16-2.19), Figure 3. Caucasian G Versus C in the grouping analysis was statistically significant, while G Versus C in the overall data was not statistically significant, suggesting that there was a significant difference between Asian and Caucasian genotypes. The results suggest that 5-HT1A (rs6295) gene polymorphism is linked with PD in Caucasian patients, and the G allele is a risk factor for PD in the Caucasian population.

**Figure 7:**
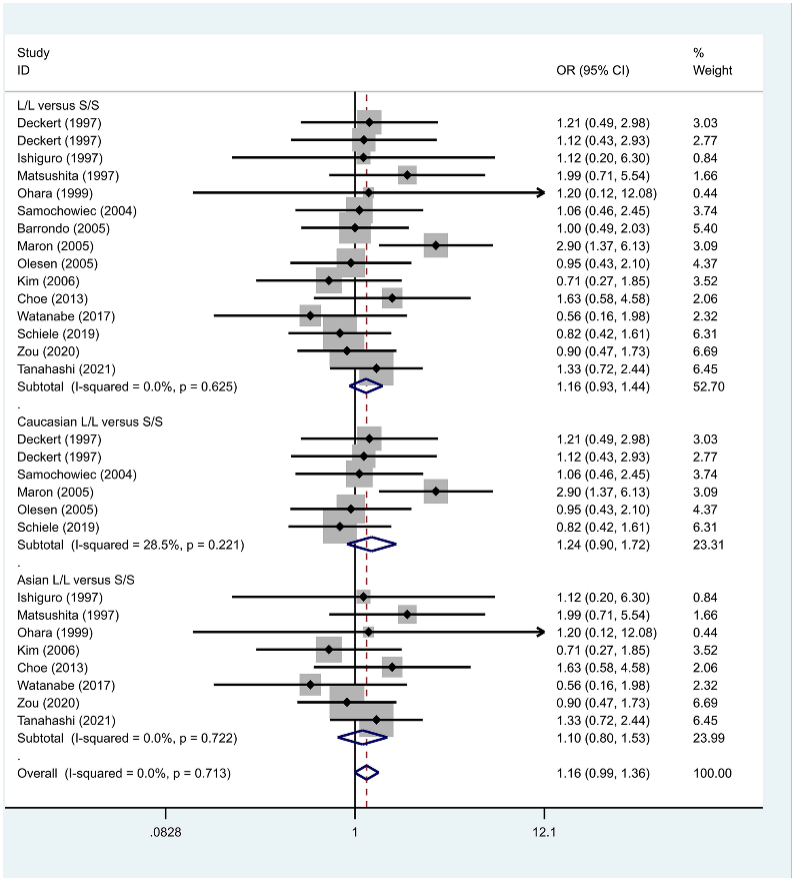
Results of the fixed-effects meta-analysis for the 5-HTTPLR genotype (L/L versus S/S) in PD and control groups.

**Figure 8:**
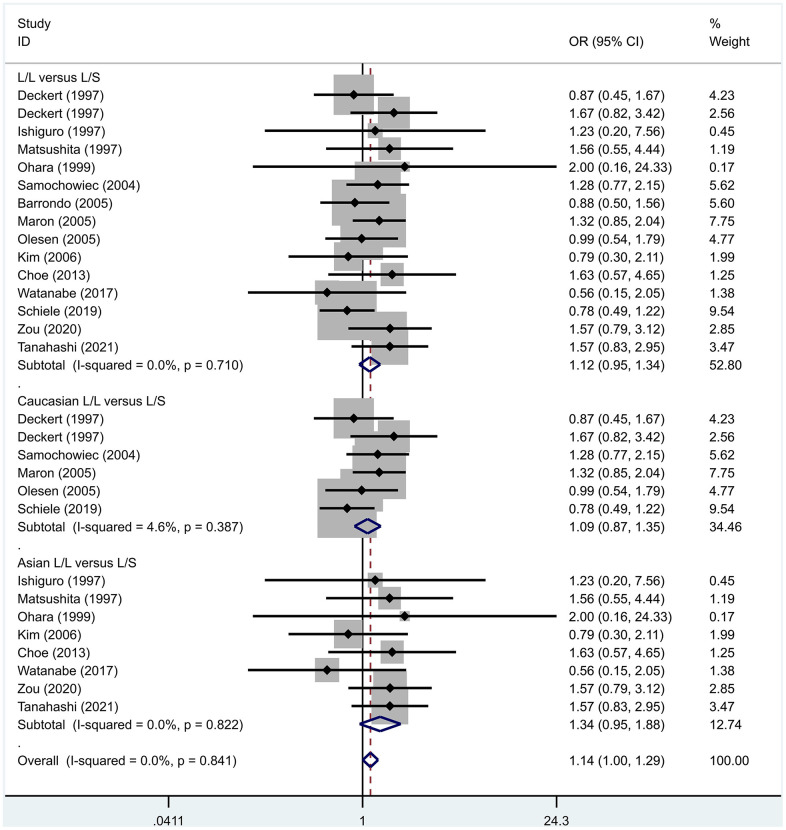
Results of the fixed-effects meta-analysis for the 5-HTTPLR genotype (L/L versus L/S) in PD and control groups.

**Figure 9:**
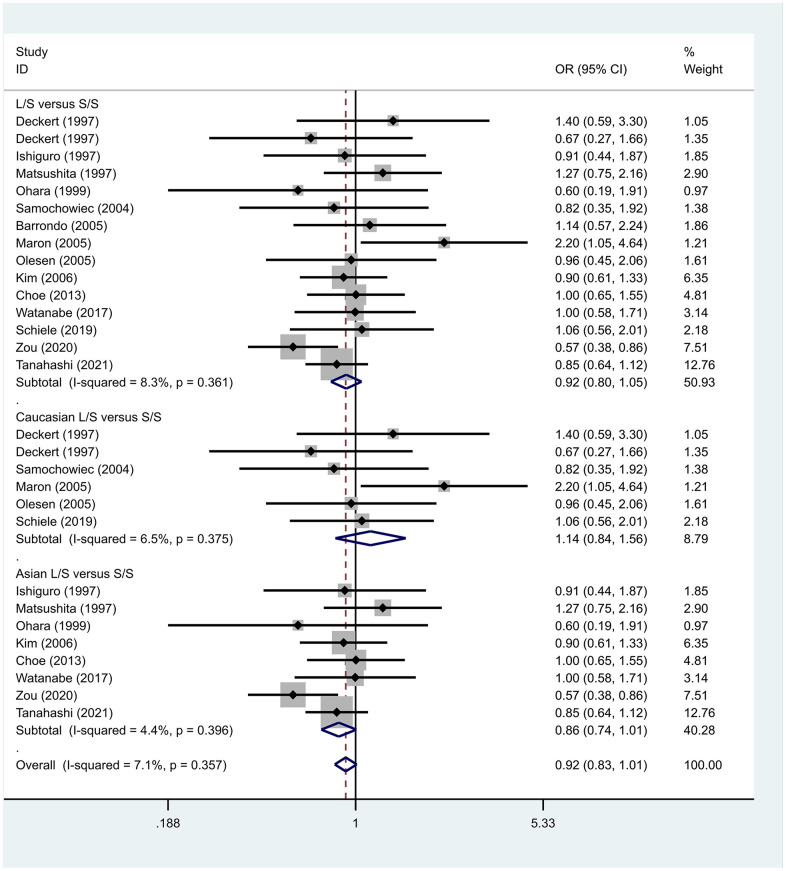
Results of the fixed-effects meta-analysis for the 5-HTTPLR genotype (L/S versus S/S) in PD and control groups.

**Figure 10:**
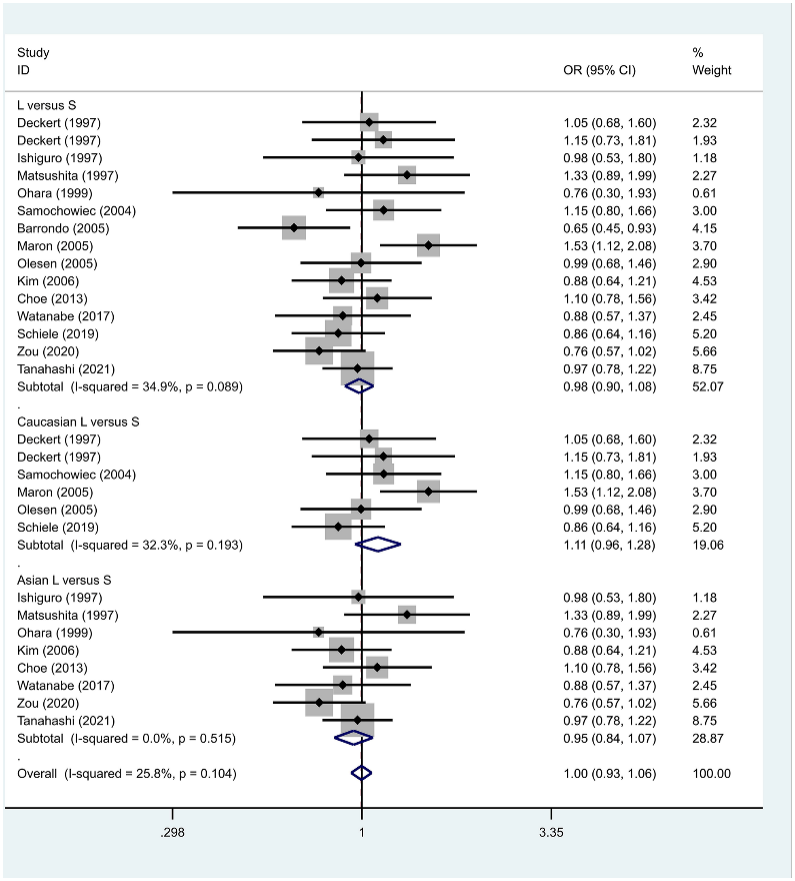
Results of the fixed-effects meta-analysis for the 5-HTTPLR genotype (L versus S) in PD and control groups.

**Figure 11:**
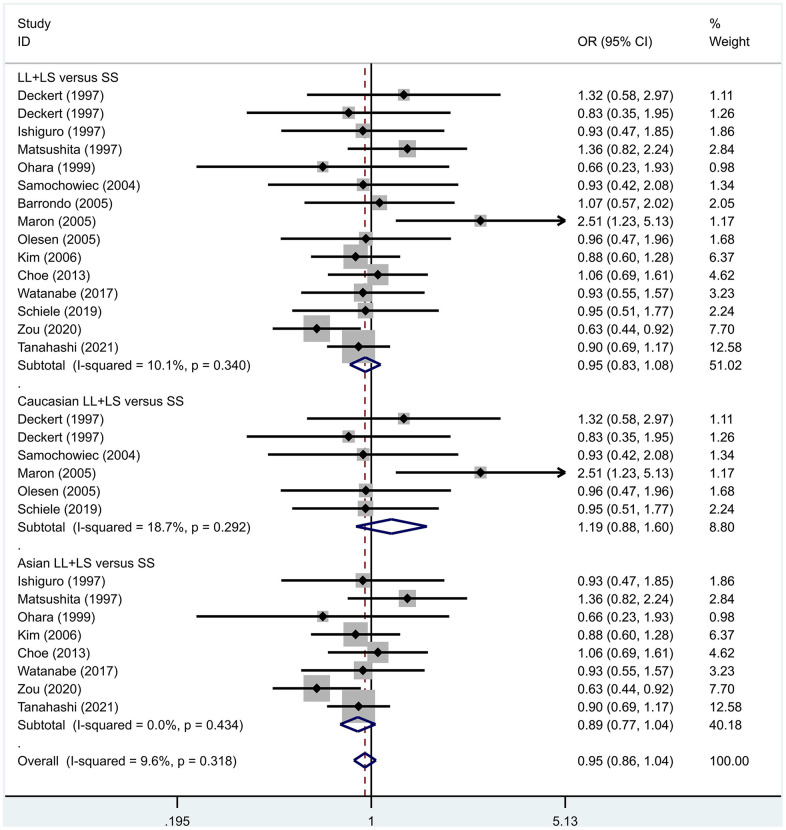
Results of the fixed-effects meta-analysis for the 5-HTTPLR genotype (LL+LS versus SS) in PD and control groups.

**Figure 12:**
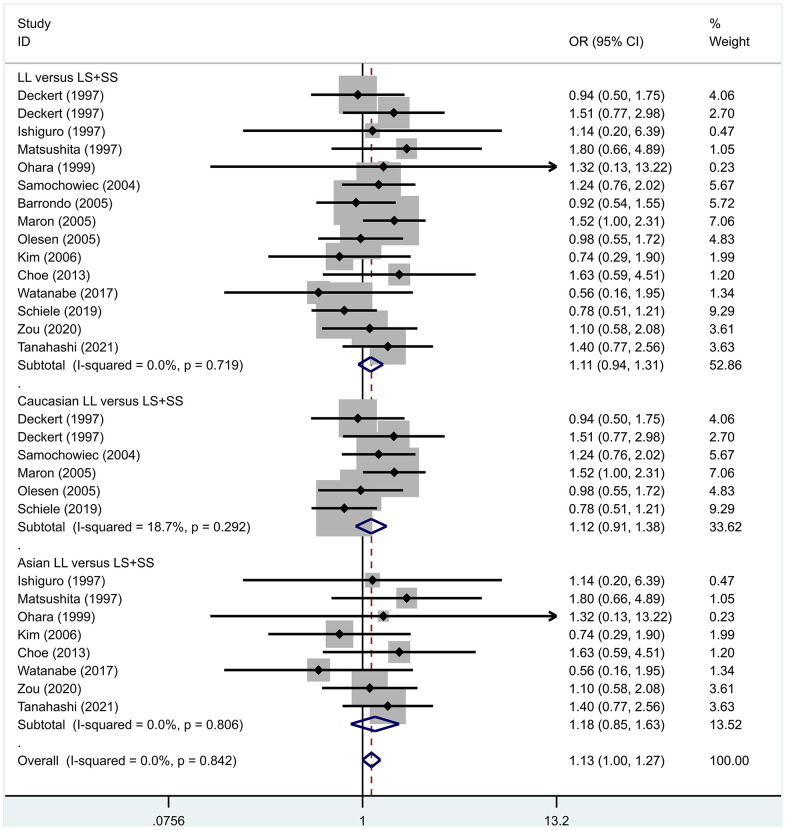
Results of the fixed-effects meta-analysis for the 5-HTTPLR genotype (LL versus LS+ SS) in PD and control groups.

Previous case-control studies showed that there was no significant difference in 5-HTTLPR gene polymorphism between patients with PD and normal subjects. To further investigate whether there was a correlation, we hypothesized that the L allele may be a susceptibility factor for PD and established a model for meta-analysis. A total of models (L/L vs. S/S, Figure 1, L/L vs. L/S, Figure 2, L/S vs. S/S, Figure 3, L vs. S, Figure 4, LL+LS vs. SS, Figure 5, LL vs. LS+SS, Figure 6) were established to test the heterogeneity of the models. In general, the heterogeneity of the model ranged from 0.00% to 34.9%, so the heterogeneity was considered small or absent, and the fixed-effect model was analyzed. No significant results were found in the analysis of all the models: L/L versus S/S: OR = 1.16, 95%CI = (0.93-1.44), Z = 1.29 P = 0.20, Figure 7; L/L versus L/S: OR = 1.13, 95% CI = (0.95-1.34), Z = 1.33, P = 0.19, Figure 8; L/S versus S/S, OR = 0.92, 95% CI = (0.80-1.06), Z = 1.20, P = 0.23, Figure 9; L versus S: OR = 0.98, 95% CI = (0.90-1.08), Z = 0.37, P = 0.71, Figure 10; LL+LS versus SS: OR = 0.95, 95%CI = (0.84-1.08), Z = 0.76, P = 0.447, Figure 11; LL versus LS+SS: OR = 1.11, 95%CI = (0.94-1.31), Z = 1.28, P = 0.200, Figure 12.

To address the differences in the results among different races, we conducted grouping analysis. The grouping criteria were the races explicitly mentioned in the study, which were divided into Caucasian and Asian race. Barrondo et al. [32] did not include information about the races. After grouping, the same model was used for grouping analysis, and the heterogeneity test was conducted on the basis of grouping, with the results ranging from 0.00% to 32.3%. The same test results showed no or small heterogeneity. The fixed effect model was used for further analysis. L/L versus S/S, OR = 1.24, 95% CI = (0.90- 1.72), Z = 1.32, P = 0.19; L/L versus L/S: OR = 1.09, 95% CI = (0.87-1.35), Z = 0.75, P = 0.46; L/S versus S/S, OR = 1.14, 95% CI = (0.84-1.56), Z = 0.83, P = 0.404; L versus S: OR = 1.11, 95% CI = (0.96 1.28), Z = 1.36, P = 0.173; LL+LS versus SS: OR = 1.17, 95%CI = (0.88-1.60), Z=1.12, P=0.27; LL versus LS+SS: OR = 1.12, 95%CI = (0.91-1.38), Z = 1.09, P = 0.278. Test results showed no significant correlation. L/L versus S/S: OR = 1.10, 95%CI = (0.80-1.53), Z = 0.59 P = 0.552; L/L versus L/S: OR = 1.34, 95%CI = (0.95-1.88), Z = 1.68, P = 0.09; L/S versus S/S, OR = 0.86, 95% CI = (0.74- 1.00), Z = 1.87, P = 0.06; L versus S: OR = 0.95, 95% CI = (0.84- 1.07), Z = 0.84, P = 0.40; LL+LS versus SS: OR=0.89, 95%CI = (0.77-1.04), Z = 1.49, P = 0.14; LL versus LS+SS: OR = 1.18, 95%CI = (0.85-1.63), Z = 1.00, P = 0.316. The test results showed no correlation. The frequency of 5-HTTPLR gene or allele is not correlated with PD.

### Sensitivity analysis

Sensitivity analysis of 5-HTIA showed that the Estimate value was 1.19 and the 95% CI was (1.10-1.30); sensitivity analysis did not affect the analysis results. Sensitivity analysis of 5-HTTPLR was that the Estimate value was 0.99 and the 95% CI was (0.94-1.05); and the results showed that the sensitivity analysis results did not affect the final analysis results.

### Publication bias

We employed the Begg’s to detect publication bias on 5-HTIA analysis results. The results showed no bias (z=1.16, P=0.24). We used Begg’s test to detect the publication bias on 5-HTTPLR analysis results. The test results showed that there was no obvious publication bias and had little influence on the analysis results (z=1.24, P=0.22).

## DISCUSSION

This is one of the first meta-analyses to investigate the link of *HTR1A* gene C-1019G polymorphisms with PD. We used 5 genotype models in case and control groups for comparative analyses. Further, the genotype-ethnic interaction model was employed to test the link of C-1019G polymorphism with PD in multiple ethnic backgrounds. The G allele or GG genotype is associated with PD in Caucasian patients. Our results showed that the 5-HTT allele frequencies and genotype distributions could not predict susceptibility to PD supporting the findings of a previous meta-analysis [37].

The 5-HT1A receptor functions not only as an auto-receptor but also as a heteroreceptor since it is found both pre-synaptically and post-synaptically. On being activated by 5-HT, the autoreceptor induces a negative feedback loop resulting in hyperpolarization and reduced firing frequency of neurons; this finally leads to lesser production and release of 5-HT. After serotonergic innervation, 5-HT exerts its effects on target neurons via the 5-HT1A heteroreceptor. Therefore, the 5-HT1A is capable of regulating the level of serotonin both globally and locally [11, 38].

Situated in the promoter region (26 base pair palindrome) of the *HTR1A*, C-1019G SNP rs6295 may affect the transcription of *HTR1A* by binding to deformed epidermal autoregulatory factor-1 (Deaf-1) and hairy enhancer of split 5 (Hes5), which are two key transcription factors. Both deaf-1 and Hes5 can specifically bind to the C-allele and inhibit 5-HT1A expression [39–42]. So, the G-1019 is associated with a higher expression of 5-HT1A autoreceptors. Increased G allele and 5-HT1A receptor binding was shown in the different brain areas based on brain imaging results [43]. As a result, the risk of developing PD is increased in patients due to higher desensitization of 5-HT1A autoreceptors, reduced firing by the raphe, and decreased serotonin level [44, 45]. However, in cells with post-synaptic expression of 5-HT1A, the function of Deaf-1 is different in presynaptic neuronal cells compared with the postsynaptic neuronal cells. Deaf-1 binds to the C allele and induces the transcription of *5-HT1A* transcription [46, 47]. The G-1019 allele may inhibit the Deaf-1-mediated transcription *of 5-HT1A* and lessen the release of 5-HT, thereby amplifying anxiety symptoms that have a lasting impact on the lives of the patients [48].

This finding was validated in human studies. It is plausible that the G/G genotype results in poor clinical outcomes, which may be attenuated by SSRI treatment which desensitizes the overexpressed autoreceptors and leads to stronger activation of 5-HT neurons. This theory was supported by Yevtushenko et al. [49] who showed that the C allele of rs6295 is linked with increased alleviation of symptoms in Caucasian patients with PD. Further, Japanese PD patients who carried the rs6295C/C responded better to paroxetine pharmacotherapy compared with non-carriers [50].

Therefore, the primary causes of reduced serotonergic neurotransmission, one of the important characteristics of PD, are the higher 5-HT1A receptors expression acting pre-synaptically and decreased postsynaptic 5-HT1A and 5-HT positive neurons [51] rs25531 is another A to G SNP, located near the 5-HTTLPR. Compared with the G-allele, rs25531 A results in the higher expression of a transporter gene via a binding site for the AP2 transcription factor [52]. Together with the 5-HTTLPR, the rs25531 leads to the L-A and L-G haplotypes known as bi-allelic or tri-allelic 5-HTTLPR. The L-G haplotype carriers show reduced lSLC6A4 levels compared with the L-A haplotype carriers [53]. However, the long G allele does not have any effect on expression similar to the short allele [54]. Importantly, previous studies concluded that 5-HTTLPR s-allele might be linked with higher amygdala reactivity and fear conditioning [55] which are the hallmarks of PD. We hypothesize that 5-HTTLPR s-allele affects the severity of symptomatic profiles and is not involved in the etiology of PD. This is supported by Strug et al. [56] who also demonstrated the bi-allelic or tri-allelic 5-HTTLPR is implicated in the severity of the symptomatic profiles and not in the etiology of PD.

One of the major limitations of this study is that the gene-gene interactions were not combined in the etiology of PD. It is established that PD is caused by many functional genes, and it is not the effect of a single nucleotide polymorphism [57]. Each gene only confers a minor risk to the disease, and the gene-gene interaction is a complex (G×G) process that affects the development of PD. Thus, future studies should investigate G×G and sub-clinical interactions (such as sex, environment factor, negative life events, and personality) to fully delineate their role in the etiology of PD.

## CONCLUSIONS

It is argued that as far as our findings are concerned, the C-1019G polymorphism correlates with the aetiology of the presence of panic disorder in Caucasians. This meta-analysis did not find any association between 5-HTT polymorphism and PD. However, subgroup analyses stratified by gender, ethnic background, severity symptoms, and other related gene polymorphisms are warranted to explore their role in the etiology of PD.

## MATERIALS AND METHODS

### Search strategy

PubMed, Web of Science, Embase, Cochrane Library, PsycINFO, and PsycARTICLE databases served as the main databases. The search was carried out for studies until July 2021, the keywords used were “5 HT1A Receptor,” “5-Hydroxytryptamine 1A Receptor”, “5-Hydroxytryptamine 1A Receptor”, “Serotonin 1A Receptor”, “5-HT1A Receptor”, “5-HT1A”, “Panic Disorders (PD)”, “Panic Attacks”, “C(-1019)G” and “rs6295”. The keywords “Serotonin Transporter Promoter (5HTTLPR)”, 5-Hydroxytryptamine Plasma Membrane Transport Serotonin Plasma Membrane Transporter Proteins, Panic Disorder, and Panic Attacks were used to research before December 14, 2021. The retrieved contents also included references to published literature.

### Inclusion criteria

1. Clear diagnostic standards (ICD-10, DSM-IV, DSM-V); 2. Detailed genotype and gene frequency data; 3. All genotypes met Hardy-Weinberg equilibrium (HWE); 4. Case-control experimental study.

### Data extraction

Basic information was extracted, including age, sex, sample size, race, result, inclusion or exclusion criteria, diagnostic criteria, assessment severity criteria, gene frequency, and allele data.

### Data analysis

Statistical software STATA 15.0 was used for analysis, as described by Shang [14]. According to different genotypes, the case group and the healthy control group were statistically analyzed for heterogeneity, the fixed effect model was selected for data with low heterogeneity, and the random effect model was selected for data with large heterogeneity for meta-analysis of dichotomous variables, bias analysis was performed to exclude the effect of publication bias on the results, and the OR value was selected for calculating the risk ratio by statistical analysis.

## Supplementary Material

Supplementary Tables
