# Supplementary material for: Association between 5-HT1A receptor C-1019G, 5-HTTLPR polymorphisms and panic disorder: a meta-analysis
Source: Aging (Albany NY). 2024 Aug 28;16(17):12293–311. doi: 10.18632/aging.206087 (PMC11424585; doi:10.18632/aging.206087)
Supplement: Supplementary Tables [file aging-16-206087-s001.pdf]

SUPPLEMENTARY TABLES

Supplementary Table 1. The characteristics of included studies explore the relationship between the 5-HT1A C-1019G polymorphism and PD.

	Sample size (Case/control)	Sex (male/female)	Age (mean±SD) (case/control)	Design	Exclusion criteria	Inclusion criteria	Diagnosis criteria	measure to assess symptom severity
Huang et al. (2004)	194 (87/107)	Not mentioned	Not mentioned 39.0±15.0	Case-control study	Drug that may affect 5-HT1A Binding was excluded.	-	DSM -IV	Not mentioned
Rothe et al. (2004)	268 (134/134)	98/169	37.3±10.8 41.0±11.0	Case-control study	-	Patients with predominant panic disorder. The controls were unrelated, anonymous blood donors.	DSM -IIIR / DSM -IV	Not mentioned
Carolina et al. (2010)	232 (107/125)	62/170	39.94±10.17	Case-control study	-	1) >90% genotype call rate; 2) minor allele frequency (MAF) >5%; 3) Hardy–Weinberg equilibrium p > 0.001. Three duplicates were assessed in the experiment and 100% concordance in genotypes was seen.	the Mini International Neuropsychiatry Interview (MINI) – Brazilian version	Not mentioned
Choi et al. (2010)	155 (94/111)	104/101	40.1±9.5, 38.3±7.3	Case-control study	1) Panic disorder patients who had comorbidity with mood disorders or other psychiatric disorders were excluded. 2) The patients who had a family history of psychiatric disorders were excluded except for anxiety disorders. The patients who had medical diseases were also excluded.	-	DSM -IV	STAI, PDSS, ASI, API, HAMA
Choe et al. (2012)	366 (194/172)	133/181	18–75	Case-control study	Exclusion criteria included any history of schizophrenia, bipolar disorder, alcohol and substance abuse or dependence, mental retardation, and current or past serious medical or neurological disorders.	-	DSM -IV	ASI-R
Takashi et al. (2017)	238 (119/119)	46/152	35.45±9.50 35.11±9.95	Case-control study	1) axis I diagnosis other than PD; 2) axis II diagnosis; 3) severe physical illness or major laboratory test abnormalities; 4) suicide risk; 5) history of substance abuse	-	DSM-IV-TR	Not mentioned
Zou et al. (2020)	464 (233/231)	Not mentioned	35.65 ± 9.77 36.96 ± 7.82	Case-control study	Patients with neurological diseases, and/or past or current	-	DSM-IV	PDSS

	episodes of MDD, generalized anxiety disorder (GAD), manic disorder, bipolar disorder, schizophrenia or any other psychiatric disorders were excluded.	

Supplementary Table 2. The characteristics of included studies explore the relationship between the 5-HTTPLR polymorphism and PD.

	Sample size (case/ control)	Sex (male/ female)	Age (mean±SD) (years)		Design	Exclusion criteria	Inclusion criteria	Diagnosis criteria	Measure to assess symptom severity
			Case	Control					
Deckert I (1997)	175 (85/90)	Not mentioned	Not mentioned		Case-control study	-	1) Patients met the DSM-III-R criteria for panic disorder. 2) The controls who had unrelated	DSM - III-R	Not mentioned
Deckert II (1997)	152 (73/79)	Not mentioned	Not mentioned		Case-control study	-	1) Patients met the DSM-III-R criteria for panic disorder. 2) The controls who had unrelated	DSM - III-R	Not mentioned
Ishiguro (1997)	216 (66/150)	30/33 70/80	27-79	25-74	Case-control study	-	1) Patients met the DSM-IV criteria for panic disorder.	DSM - III-R	Not mentioned
Matsushita (1997)	299 (86/213)	54/32 96/117	37.0 ± 12.1	37.7 ± 13.1	Case-control study	-	1) Patients met the DSM-III-R criteria for panic disorder. 2) The controls who had unrelated	DSM - III-R	Not mentioned
Ohara (1999)	133 (27/106)	11/16 47/59	38.7±10.5	35.6±11.6	Case-control study	-	1) Patients met the DSM-IV criteria for panic disorder. 2) The controls had no history of psychiatric diseases or psychotropic medication in the past.	DSM -IV	Not mentioned
Samochowiec (2004)	357 (191 /166)	54 /148	38.7±11.8	35.9±14.3	Case-control study	1) Subjects with a history of a primary major psychiatric disorder or substance dependence other than nicotine dependence were excluded	-	ICD-10	Not mentioned
Barrondo (2005)	238 (119/119)	28/64 67/107	35.87±12.38	38.40 ±8.94	Case-control study	-	1) Patients met the DSM-IV criteria for panic disorder.	DSM -IV	Not mentioned
Maron (2005)	373 (158/215)	32/ 126 56/159	38.0±12.9	39.8±13.0	Case-control study	-	1) Patients met the DSM-IV criteria for panic disorder. 2) Only healthy subjects without personal or family history of psychiatric disorders among first-degree relatives were included.	DSM -IV	Not mentioned
Olesen (2005)	212 (104/108)	28/76 30/78	Not mentioned		Case-control study	-	1) Patients met the DSM-IV criteria for panic disorder. 2) Patients were unrelated Scandinavians with a Caucasian genetic back-ground.	DSM -IV	Not mentioned
Kim (2006)	471 (244/227)	143/101 102 /125	36.1 ± 9.0	33.1 ± 9.1	Case-control study	-	1) Patients met the DSM-IV criteria for panic disorder. 2) Patients with comorbid major depressive disorder were also included.	DSM -IV	Not mentioned

Choe (2013)	357 (191 /166)	Not mentioned	18–75		Case-control study	1) any history of schizophrenia, bipolar disorder alcohol and substance abuse or dependence, mental retardation, and current or past serious medical or neurological disorders	-	DSM -IV	Not mentioned
Watanabe (2017)	238 (119/119)	43/76 43/76	35.45±9.50	35.11±9.95	Case-control study	1) axis I diagnosis other than PD; 2) axis II diagnosis; 3) severe physical illness or major laboratory test abnormalities; 4) suicide risk; 5) history of substance abuse.	-	DSM -IV-TR	Not mentioned
Schiele (2019)	287 (109/536)	40/ 69 153/383	36.2±10.9	25.2±5.6	Case-control study	1) past or present diagnosis of any DSM-IV axis I disorder as ascertained by the Mini International Psychiatric Interview or severe neurological or somatic disorders.	-	DSM -IV	Not mentioned
Zou (2020)	464 (233 / 231)	92/141 98 /133	35.65±9.77	36.96±7.82	Case-control study	-	1) All subjects were free of acute or chronic somatic disorders. 2) All patients were free of antidepressants or other psychotropic medications intake within 2 weeks before their examination.	DSM -IV	PDSS
Tanahashi (2021)	955 (515/440)	148/367 146/294	38.72±10.26	37.83±10.82	Case-control study	1) subjects who had lost consciousness due to major physical or neurological disorders or had alcohol abuse, substance abuse, or head trauma were excluded.	-	DSM -IV	Not mentioned
